# Invisible Agency in the Search for Healing: Patient and Family Roles in the Care of Hard‐to‐Heal Wounds in Primary Healthcare

**DOI:** 10.1111/iwj.70970

**Published:** 2026-06-04

**Authors:** Henna Odisho, Malin Karlberg‐Traav, Agneta Anderzen‐Carlsson, Georgina Gethin, Dimitri Beeckman

**Affiliations:** ^1^ Swedish Centre for Skin and Wound Research (SCENTR), School of Health Sciences, Faculty of Medicine and Health Örebro University Örebro Sweden; ^2^ Post Graduate School for Integrated Care Örebro University Örebro Sweden; ^3^ University Health Care Research Centre, Faculty of Medicine and Health Örebro University Örebro Sweden; ^4^ School of Nursing and Midwifery University of Galway Galway Ireland; ^5^ Alliance for Research and Innovation in Wounds University of Galway Galway Ireland; ^6^ Skin Integrity Research Group (SKINT), University Centre for Nursing and Midwifery, Department of Public Health and Primary Care Ghent University Ghent Belgium

**Keywords:** family support, patient‐centred care, self‐management, skin ulcer, wound healing

## Abstract

This study aimed to explore how persons living with a hard‐to‐heal wound and their family members experience care. The inclusion criteria for patients were wounds that had persisted for more than 6 weeks or hard‐to‐heal wounds that had recently healed. The study included 16 participants (13 patients and 3 family members) from primary healthcare services in Örebro County, Sweden. The interview data were transcribed and analysed using reflexive thematic analysis. One overarching theme was generated: Navigating an uncertain path towards healing, along with three subthemes: (1) Striving to be an active agent, (2) Being part of collaboration efforts and (3) Being a bystander in the search for the right treatment. Together, these themes illustrate how patients and family members engaged in an uncertain care process as they sought to understand the condition and manage care in everyday life. These everyday efforts reflected forms of invisible agency, as participants did not always recognise them as meaningful contributions to wound care. The findings highlight the importance of person‐centred approaches that recognise and value patients' and family members' everyday contributions to wound care and support self‐management through partnerships among patients, family members and HCPs.

## 1 Introduction

Persons with hard‐to‐heal wounds experience a significant biopsychosocial burden that affects their quality of life. Family members are also affected, facing psychological, social and financial strain [1, 2]. Hard‐to‐heal wounds are defined as those that do not achieve complete closure within 6 weeks [3]. They are characterised by impaired progression through the normal healing process, often influenced by underlying conditions such as venous insufficiency, arterial disease, prolonged pressure, diabetes‐related neuropathy, or infection [4]. Since different wound aetiologies affect healing trajectories and require specific treatment and prevention strategies, a thorough clinical assessment is essential [5, 6]. Such an assessment should not only guide effective care but also consider how the wound and its treatment affect the person's daily life, functioning and well‐being [6].

The chronic nature of hard‐to‐heal wounds often leads to prolonged, demanding care trajectories, necessitating ongoing treatment and monitoring and placing greater demands on access to care and coordination [7]. The incidence of hard‐to‐heal wounds is expected to rise, driven by an ageing population and an increasing prevalence of risk factors such as obesity and diabetes [8]. Globally, the number of people with diabetes is projected to more than double by 2050 [9]. At any given time, approximately 6% of people with diabetes live with an active foot ulcer [10] and wounds of mixed aetiologies affect about 2.2 per 1000 in Western societies [11]. Primary healthcare (PHC) plays a central role in supporting patients and their families through ongoing wound care, care coordination across services and preventive efforts integrated into everyday care [12].

Approximately 40 000 people in Sweden live with hard‐to‐heal wounds, and recent findings indicate that almost half lack a documented diagnosis [13]. In Sweden, wound care is primarily managed within PHC, which includes both regional and municipal services that collaborate with specialist care to ensure coordinated care [14, 15]. Alongside formal care, patients and their family members often manage aspects of wound care in their daily lives [16]. In some cases, care is managed without direct involvement of healthcare services, and family members may be involved without structured support or guidance [17, 18].

Over recent decades, research on person‐centred care (PCC) has expanded considerably, reflecting growing recognition of the limitations of traditional care models in meeting patients' needs [19, 20]. Person‐centred care builds on recognising the person and understanding their values, preferences and lived experiences [21]. It emphasises partnership, with patients and, when appropriate, family members actively involved in care through shared decision‐making and ongoing dialogue. In PCC, supporting self‐management is an important aspect of care [22]. Self‐management involves a person actively managing the medical, emotional and social consequences of living with a long‐term health condition—often in collaboration with family members and healthcare professionals (HCPs). It goes beyond self‐care tasks and includes a person's capacity to participate, make decisions and solve problems in daily life [23]. However, the ways in which patients and family members contribute to care are not always recognised within the care process [24].

A systematic review found that home‐based wound care training strengthens patients' and family members' roles in wound care and may improve clinical outcomes [25]. However, advice on lifestyle‐related aspects of wound care has not been associated with faster healing, suggesting variability in how such guidance is implemented in daily life [26]. Research on person‐centred self‐management support has primarily focused on specific wound aetiologies [27], whereas wound care research more broadly has prioritised clinical outcomes, often overlooking patients' and family members' experiences and priorities [18, 28]. As a result, there is limited understanding of how continuous, person‐centred self‐management support can be organised and evaluated within wound care in PHC [27, 29, 30]. In practice, patients may find it challenging to understand what is expected of them and to self‐manage confidently. Their everyday struggles may also remain unrecognised in clinical practice [31]. Relational aspects of care may further limit patients' involvement. Patients may face structural and interpersonal barriers in care encounters that limit their involvement in decision‐making, reduce their autonomy and increase their vulnerability [32]. Studies in wound care show that patients feel unheard and unsupported when their experiences are dismissed or when care goals differ from their own [31, 33]. However, when patients are recognised and met as a person in a caring relationship with HCPs, they can feel supported [31]. A deeper understanding of patients' and family members' experiences is needed to inform more responsive and person‐centred approaches to wound care in PHC. Therefore, the aim of the study was to explore how persons living with a hard‐to‐heal wound, as well as their family members, experience care.

## 2 Materials and Methods

### 2.1 Study Design

An experiential qualitative design was adopted to capture and describe how participants experienced and made sense of the phenomenon under study [34]. Care was conceptualised broadly to encompass participants' experiences of professional care across the care continuum, as well as their own and their family members' actions, reflections and self‐management efforts related to the wound. This study formed the first phase of a comprehensive mixed‐methods research project conducted as part of a doctoral dissertation in nursing science.

### 2.2 Setting and Participants

The study included patients and family members recruited from both regional and municipal PHC services in urban and rural areas of Örebro County, Sweden, allowing for sociodemographic variation among participants. The inclusion criteria for patients were wounds that had persisted for at least 6 weeks or hard‐to‐heal wounds that had healed within the past year. Family members involved in wound care were also eligible to participate. Purposive sampling was used, and HCPs informed patients with diverse wound aetiologies, thereby promoting heterogeneity in the sample. Patients received verbal information and an information letter during follow‐up appointments and were encouraged to share the letter with family members.

The first author contacted individuals who had expressed interest in participating to arrange an interview. Following the interview, with participants' consent, the first author accompanied them to a scheduled wound‐dressing appointment, during which wound photographs were taken. The first author had no clinical role in the care provided during these encounters. The photographs were collected as part of the demographic data to provide contextual information about wound type, particularly when participants were unsure of the cause of their wound. This also supported purposive sampling by ensuring variation in wound aetiology across the sample.

### 2.3 Data Generation

Individual interviews were conducted between October 2024 and April 2025 and comprised 12 face‐to‐face and four virtual interviews. Fifteen interviews were conducted by the first author (HO), a registered nurse and district nurse with 5 years of experience in wound care in PHC and a PhD candidate in nursing. The second author (MKT), a registered nurse with a PhD in nursing and extensive experience in qualitative research, conducted one interview. The authors had no prior clinical relationship with the participants and had not been involved in their care. A semi‐structured interview guide was used and organised around four domains: challenges in wound care; wound‐care strategies; the role of family members; and overall experiences of wound care. The research team developed the interview guide through team discussions, drawing on the group's combined clinical and research expertise. The guide consisted of open‐ended questions and was piloted in the first interview; as no revisions were required, this interview was included in the analysis. The interview questions are presented in Box 1.

BOX 1 **Interview guide.**

**Table:** 

Can you tell me how your wound care is carried out?
Can you tell me how you take care of your wound yourself?
What do you think affects your ability to care for your wound during this time?
Do you have someone close to you who helps you?
In what way does that person assist you?
Who is responsible for your wound care?
How do you think it works?
What works well?
Is there anything you think could be improved?

The same interview guide was used, but some questions were adapted for use with family members, who were asked about how the patient's wound care was managed, how they assisted in care, how their support affected the patient's ability to manage the condition and factors influencing their own capacity to provide support. Probing questions were used to encourage more in‐depth responses. The interviews lasted 16–76 min (mean = 40 min), were audio‐recorded and transcribed verbatim.

### 2.4 Data Analysis

Braun and Clarke's reflexive thematic analysis was followed to identify patterns of shared meaning within the dataset [35, 36]. A pragmatic stance informed the analysis, focusing on how participants made sense of and responded to their experiences within everyday life and the care process, and how these experiences can inform PHC practice [37]. Analysis was conducted using NVivo 14 software [38]. The first phase involved familiarisation, with the first author reading and re‐reading the transcripts to gain a deep understanding of the material. Reflexive notes were taken throughout, and active engagement was maintained by asking questions about meaning, relevance and alignment with the study aim. The second phase involved systematic coding of the entire dataset, including all content relevant to the research aim. Coding was conducted at both the semantic and latent levels to capture participants' explicit accounts and underlying meanings. The first author performed initial and second‐round coding and the research team reviewed the code content through discussions. Guided by the research aim, the codes were sorted into potential themes. These initial themes were discussed and refined in dialogue with the research team. The first author revisited the transcripts and reflexive notes to reconnect with the data and clarify interpretations during theme review. Finally, themes were defined and named, and the report was written. The process proceeded iteratively, moving back and forth between phases [35, 36].

### 2.5 Researcher Reflexivity

Reflexivity involves ongoing consideration of how researchers' backgrounds, experiences, assumptions, positions and contextual understandings may influence the research process and interpretation of findings [39]. The research team brought diverse clinical and academic expertise, which influenced data interpretation and contributed to critical discussions throughout the research process. Most interviews were conducted, and the analysis was led by the first author, whose insider position in PHC and experience in wound care provided familiarity with the organisational context and the care practices described by participants. This position necessitated ongoing reflexive work to avoid assuming shared meanings or overlooking aspects of care that might appear routine. Reflexive notes were recorded after each interview and throughout the analytic process, including reflections on assumptions, pre‐understandings and emerging interpretations. Reflexivity was further supported by ongoing discussions within the research team, where interpretations and pre‐understandings were critically examined in relation to participants' accounts.

### 2.6 Ethical Considerations

The study was approved by the Swedish Ethical Review Authority (Ref. No. 2024–04063‐01). The principles outlined in the Declaration of Helsinki were followed to protect the rights and safety of all participants [40]. All participants received written and verbal information about the study, and written informed consent was obtained before the interviews. When patients and family members who knew each other participated, it was emphasised that information shared in individual interviews would not be disclosed to the other party. All data were securely stored on the university's research server to safeguard confidentiality and were accessible only to the research team.

## 3 Results

A total of 16 participants were included: 13 patients and 3 family members. Two family members were related to participating patients, whereas one participated independently. Patient ages ranged from 36 to 90 years (mean = 68), and family members' ages ranged from 37 to 63 years (mean not meaningful, as the sample size was only 3). All family members were women, either spouses or adult children. Among the patient participants, nine were men and four were women. Educational backgrounds varied: half of the participants held a university degree, four had completed secondary education and four had completed compulsory schooling. Participants presented with wounds of various aetiologies, including venous leg ulcers, pressure ulcers, diabetic foot ulcers, trauma‐related wounds, infections delaying healing and ulcers associated with other underlying conditions. The experiences of hard‐to‐heal wounds ranged from single episodes resolving within months to wounds persisting for over a decade. Participants had received care across different parts of the healthcare system, including municipal or regional PHC and specialist care.

**FIGURE 1:**
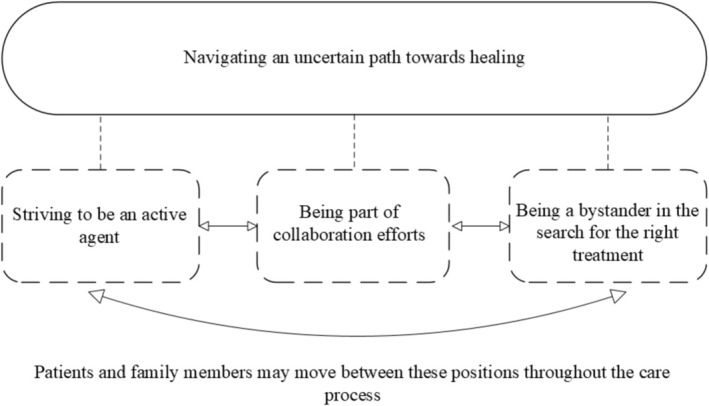
Overview of the overarching theme and subthemes. The figure illustrates wound care as an uncertain process in which patients may move between different ways of engaging in care. These positions are dynamic and situational rather than linear stages. Family members remain by the patient's side throughout the care journey.

The analysis generated an overarching theme, ‘Navigating an uncertain path towards healing’, that interprets the underlying meaning of participants' experiences and captures the unpredictability and endeavours reflected throughout the dataset. The care and treatment process rarely followed a predictable trajectory, as various factors influenced its course. At the same time, patient participants' experiences were marked by constant striving; they sought to manage the situation and maintain hope for wound healing despite ongoing uncertainty. Family members experienced similar uncertainty, shaped by their position on the sidelines of care. This overarching theme was constructed from three interrelated subthemes: ‘Striving to be an active agent’, ‘Being part of collaboration efforts’ and ‘Being a bystander in the search for the right treatment’ (Figure 1). These subthemes represent situational and dynamic ways of engaging in care that patients and family members moved between throughout the care process.

### 3.1 Striving to Be an Active Agent

This subtheme captures how patient participants navigated wound care at home and the conditions that enabled or constrained their efforts to take an active role.

Several participants managed their wound dressings at home at different points—some months before seeking PHC, and others when the dressings came loose. Some described doing their best with the knowledge they had. Later, some learned from HCPs that the cleaning or dressing methods they had used did not align with their wounds' requirements, which made them aware of their limited knowledge and reinforced the sense that practical wound care might be beyond what they could safely manage on their own. Inconsistent advice from different HCPs about what to do when a dressing came loose also contributed to insecurity.

In contrast, some participants described feeling confident in performing dressing changes after observing HCPs, copying their techniques and receiving reassurance during follow‐up visits. They described wound care as a shared responsibility, with them managing daily care and monitoring while HCPs provided clinical oversight.

If I notice anything, I will inform the PHC centre the next day, and we can then schedule an additional visit if needed. That is pretty much how the nurse and I have agreed to manage things. (P16)

This shared responsibility and clarity about when to seek help if the wound worsens provided a sense of security. Still, some described taking over responsibility too early, which led to deterioration and a return to previous care. Even so, they hoped to manage independently again once the wound had improved. Participants emphasised the importance of having appropriate dressings at home, and some expressed concern that limited access to suitable or affordable equipment might reduce the effectiveness of home care.

Although several equated wound care with dressing changes and some felt unable to contribute if they could not perform them, their narratives revealed active engagement in other ways. Participants described a range of strategies shaped by vigilance, caution, or underlying concern about wound deterioration. These included protecting the wound during showers using assistive devices or improvised solutions, avoiding full‐body showers, adjusting daily activities to reduce pressure or the risk of accidental injury, monitoring wound changes with photographs and checking body temperature to detect signs of infection. Participants who showered before appointments described exposing the wound as painful, and some worried that leaving it uncovered for too long could cause deterioration. These practices demonstrate that participants engaged in protective and preventive care, sometimes with uncertainty and that these actions were not always recognised by themselves as care.

Beyond these protective efforts, participants described striving to maintain their usual activities, though often with adjustments. They emphasised the importance of independence and a sense of control over their daily lives, yet the wound and its treatment disrupted this at times. Some participants described prioritising other aspects of everyday life over treatment recommendations, leading them to occasionally deviate from the prescribed care plan despite HCP advice.

Pain restricted some participants' agency. Opioid‐based pain medication reduced the ability to drive and increased dependence on others, whereas insufficient relief reduced participants' capacity to manage daily tasks. Some felt compelled to take more medication than prescribed to cope, which in some cases led to complications requiring hospitalisation. These experiences evoked fear or left participants feeling they had to endure pain even when pain medication was insufficient.

Compression therapy introduced additional demands. Some participants found restrictive compression uncomfortable or disruptive, yet felt they had no choice but to endure it, believing it was essential for healing. Others described improved self‐management after receiving customised compression solutions, allowing them to wear regular shoes and shower on their own schedule. Although this improved comfort and adherence, practical limitations, such as receiving only one pair, still caused stress and restricted daily life.

Self‐care strategies to support healing rarely emerged spontaneously. When asked to reflect more broadly on wound care, some still could not identify any personal actions related to it

I: Do you do anything else to support wound healing?

P: No, nothing consciously, at least. I am unsure what I should do. (P14)

This suggests a limited understanding of how their actions can impact wound healing or prevent deterioration, thereby limiting their sense of agency.

Some participants emphasised the importance of having a clear self‐care plan. Although some had received such guidance and valued it, others reported lacking this support. Participants described trying to improve their nutrition and engaging in activities such as physical activity, pressure offloading, leg elevation and skin protection to support wound healing. However, pain, wound location, difficulties with shoes, challenges finding suitable activities and uncertainty about whether physical activity might worsen the wound sometimes limited engagement. Some therefore sought advice from HCPs, while others reduced their activity independently. Both patients and family members emphasised the importance of adhering to home treatment recommendations also after healing, which they described as essential to prevent deterioration.

### 3.2 Being Part of Collaboration Efforts

Participants highlighted relationships and supportive interactions with HCPs, which made a difficult situation more manageable. They described developing familiarity and trust with HCPs over time. Some even described the relationship as ‘like meeting a friend’, and said that appointments became something they looked forward to—not because of the wound care, but because of the people. Continuity strengthened this sense of security; however, when continuity was not possible, some described feeling reassured when all HCPs adhered to the same care plan and provided consistent treatment.

HCPs were also described as conveying hope by highlighting signs of improvement, and some participants noted that being involved in wound care procedures increased their knowledge.

The nurse showed me the wound through a magnifying tool and explained what was happening. (P4).

Having HCPs identify changes in the wound and highlight even minor signs of progress fostered hope and strengthened their belief in healing. Some felt reassured when HCPs were honest about the time required for healing, whereas others continued to struggle to understand why healing was so slow, and some sought additional information from HCPs.

The organisation of care shaped collaboration. Participants generally understood who was responsible for their care, often identifying nurses or assistant nurses as primarily responsible for wound care. Others reported feeling safer when dressing changes performed by assistant nurses were overseen or followed up by a registered nurse. Medical doctors were described as becoming involved when needed—for prescriptions, diagnostics, or initiating compression therapy—and their involvement could reinforce trust in the care process.

Some participants described practical challenges in managing appointments and travelling for treatment. When care was provided at home, participants described how staff shortages, limited continuity and unclear communication could make care feel inconsistent and disrupt everyday routines. Some described learning to navigate the system over time, including when and where to seek contact. Coordinated appointments and collaboration between healthcare providers were described as reducing logistical burdens and making care feel more manageable.

As healing progressed, longer intervals between dressing changes reduced the need for frequent visits and made everyday life easier to manage. Some participants also described how HCPs addressed underlying health problems that had affected their well‐being prior to the wound. For some, this fostered gratitude, as the wound prompted the identification and management of health issues that might otherwise have gone unaddressed.

Conversely, a lack of follow‐up, unexplained changes in staffing, or inconsistent communication created insecurity in the collaboration between patients and HCPs. Participants called for stronger collaboration among team members to improve care coordination. Some participants learned which professionals were involved only by reviewing their medical records. Limited continuity meant they had to repeat their story to multiple HCPs unfamiliar with their case, diminishing confidence in the care process.

Family members were also involved and provided important, though varied, forms of support. As described by both patients and family members, their involvement depended on practical availability and the patient's need for assistance; in this dataset, it was typically sporadic rather than routine. Most family members did not participate in dressing changes due to discomfort, lack of knowledge, or, in some cases, because patients felt uncomfortable receiving such help from family members. Instead, as described by both patients and family members, their support included assisting with compression, inspecting the wound, offering advice, organising transportation, or helping interpret changes in the wound. Although family members contributed in multiple ways, some had difficulty recognising or articulating the significance of their involvement, reflecting uncertainty about their role rather than a lack of commitment.

The way I help is by talking about it and sometimes checking it. And I'm not sure it really makes much of a difference. (P12, family member)

Patients described family members accompanying them to appointments when needed and providing logistical and emotional support, which was valuable in daily life and for safeguarding patients' interests during care encounters. However, family members' ability to attend healthcare visits was often constrained by work commitments and limited appointment availability. Care information was often obtained indirectly through the patient or medical records, although some family members preferred direct communication with HCPs for reassurance. A family member assisting with dressing changes felt more reassured when they could confirm the wound changes with HCPs and receive guidance on interpretation and next steps. Family members' support in recognising and responding to signs of deterioration was sometimes crucial—even lifesaving—as some family members and patient participants described. Occasionally, family members and the patient held differing opinions about whether the wound had worsened and whether it was time to seek medical care.

Yes, and then you're left with this—when half the foot is completely red, and it doesn't look right. I said: It really doesn't look good, but the patient says HCPs said it looked fine the last time they changed the dressing. (P3, family member).

This disagreement led to the family member's resignation, as they felt compelled to withdraw. Furthermore, uncertainty about interpreting wound changes and the discomfort associated with the wound created insecurity among family members, who felt that greater knowledge would have made assessment easier. Patient participants viewed family members with medical expertise as sources of advice regarding wound management and determining when to seek care. However, this responsibility could feel overwhelming; in retrospect, a family member with medical expertise believed they had taken on too much.

Family members described a lack of clarity about the care process, including who was responsible for different aspects of care, which professionals were involved and whether a formal care plan guided wound management. This made it difficult for them to judge whether the care or HCP collaboration was functioning as intended. Some family members felt that essential follow‐up had been missed due to the involvement of multiple caregivers without a clear overall responsibility. There was also uncertainty about whether delays in receiving appropriate care could be attributable to the patient's initial refusal of particular supports. Some patient participants also expressed uncertainty about whether their wound care had been adequately assessed or coordinated across specialists. This lack of transparency in the collaborative process diminished their sense of orientation and their confidence that the appropriate services or specialists were engaged.

### 3.3 Being a Bystander in the Search for the Right Treatment

Across interviews, participants' descriptions of parts of the care trajectory suggested that they experienced the process from a bystander perspective. They generally expressed satisfaction with the care they received, describing HCPs as supportive, well‐intentioned and doing their best. At the same time, many described the treatment process as a trial‐and‐error process that requires HCPs to spend time identifying a suitable approach. Some participants acknowledged the challenges of treating hard‐to‐heal wounds. During this search phase, they relied on HCPs' clinical expertise to guide decisions. For some, stepping back thus felt natural. ‘I am not someone who really understands these things’. (P6). This reflected not only a perceived lack of knowledge about wound treatment but also the notion that being on the sidelines of decision‐making did not always carry negative implications.

For some, the trial‐and‐error phase was prolonged and marked by recurrent setbacks, such as wound deterioration, infections or sepsis. Although participants expressed gratitude that healthcare eventually managed these complications, the experience left lasting impressions and a fear of recurrence or further complications. A limited understanding of why the wound failed to heal or why infections recurred made it difficult; some began to doubt their own body's ability to heal, and at times questioned whether something fundamental was being overlooked. At times, participants wondered whether the right decisions were being made or whether more effective alternatives existed—sometimes because of differing opinions among HCPs. These concerns became clearer in hindsight: Some described realising the limitations of their earlier care only once HCPs with greater wound‐care expertise became involved, and the wound improved markedly with their methods. This contrast sharpened their sense that earlier access to specialised expertise might have altered the course of healing. Furthermore, some participants wished they had been more involved in the process and wondered whether they could have done more themselves to identify the appropriate treatment. Both patients and family members described how family members encouraged patients to participate in treatment‐related decisions by urging them to speak up when they felt something was not right or when their concerns were being dismissed. In contrast, other patient participants felt hopeless, believing both themselves and HCPs had already tried all options.

Wound treatment could significantly affect well‐being, and some participants felt they had no real choice but to endure complicated or painful procedures, as these were necessary for the wound. Pain during dressing changes occurred at times; some reported that it was particularly severe during infections. For some, the pain was so overwhelming that, despite receiving a local anaesthetic, they struggled to follow what HCPs were saying or deciding during the appointment. One participant described refusing treatment because the pain was unbearable. The refusal was not readily accepted; instead, HCPs continued to urge them to proceed. Despite this, the participant maintained their decision to refuse treatment, although this led to feelings of not having done everything possible for their recovery.

For some, being a bystander was a role imposed when their needs or concerns about treatment went unheard or were given lower priority, leaving them feeling unable to progress in daily life. Participants with severe daily pain emphasised that this was an issue HCPs needed to address, as it was otherwise unmanageable. One participant said that ‘the pain takes over more than anything else’ (P15). Not being heard led to frustration and a reduced sense of agency in the care process, and some eventually resigned. Others reflected on whether they should have spoken up more during the decision‐making process but found it difficult to do so at the time, as treatment decisions were seen as within the professionals' domain.

You feel you must leave it to the doctors and nurses—it is their job, and you do not feel it is your place to interfere (P5).

In contrast, others described moments where they actively asserted themselves after feeling sidelined in decision‐making. When HCPs pushed for decisions that did not align with their own views, or when they felt their condition was not taken seriously, they spoke up—drawing on their sense of knowing their bodies best or on their awareness of their rights as patients.

A lack of clear information further reinforced the bystander role. Some participants sought to deepen their understanding by asking questions or reviewing their medical records, but medical terminology remained a barrier to fully understanding their condition or treatment. Some wished they had received more precise explanations throughout the process. Some family members attempted to fill these gaps by searching online or consulting the patient's medical record to check whether the care and treatment aligned with current guidelines. Even when it did, some still worried as the wound deteriorated, fearing further complications. This shows how family members sought ways to stay informed when they could not participate in appointments, relying on indirect sources to understand the care process.

Furthermore, patients' and family members' accounts revealed aspects of care they considered important and what they believed would meaningfully strengthen future wound care. These reflections often emerged after opportunities to influence care had passed and therefore are placed under this theme See Table 1.

**TABLE 1:** Participants' perspectives on strategies to improve wound care and on important considerations when selecting the most appropriate treatment.

Perspective	Key areas identified	Implications for practice
Patients Patients Family members Patients Family members	Collaboration and recognition Support for self‐management Support for self‐management Clinical management and prevention Clinical management and prevention	Use shared care planning across professions and include the patient Adapt treatment plans to individual patient needs and preferences Learn from previous failures by including and asking patients about their experiences Provide early access to home‐based treatment options and assistive devices that support independence Clearly communicate what patients can do at home to support healing Provide individualised compression options to support adherence Support patient understanding of compression therapy Explore how the wound affects the patient's daily life Tailor support based on everyday challenges Ensure access to a wound care specialist at the PHC centre, or prompt referral elsewhere if unavailable Ensure timely medical review when healing is delayed Assess underlying factors affecting wound healing Establish a clear ‘plan B’ if healing stalls Even if fewer dressing changes are medically optimal, consider the patient's experience of odour Address risk factors such as oedema before wounds develop Ensure infections are adequately identified and followed up on Allow sufficient time to evaluate treatment effect

## 4 Discussion

Understanding patients' and family members' experiences of wound care can help identify where PCC breaks down in everyday practice and clarify what needs to change to support more consistent PCC in primary healthcare. The findings illustrate the ongoing work required to navigate a wound care trajectory characterised by uncertainty. They further highlight the agency demonstrated by persons living with hard‐to‐heal wounds and their family members through everyday efforts to manage care and adapt to changing circumstances throughout the care process. These forms of agency require greater recognition within healthcare practice [24].

However, participants did not always recognise these efforts as meaningful contributions to care or as forms of self‐management. This underlines the notion of an invisible agency in wound care. The findings suggest that features of the care process, together with participants' confidence in themselves and their perceived knowledge, shape not only what patients and family members can do but also how they come to understand their roles in care. The participants' everyday efforts tended to remain invisible in situations characterised by inconsistent advice or the absence of a shared care plan, leaving them unsure whether their actions were appropriate or safe. Moreover, without a language to frame efforts to understand and influence care, support healing and prevent deterioration as forms of care, participants did not recognise these efforts as such. When feedback and support from HCPs were present, however, invisible agency became more visible, strengthening participants' confidence in managing wound care. These findings indicate that when care processes, roles and expectations are made explicit, patients and family members are better able to recognise their own contributions and to articulate when support is needed. In this sense, making agency visible is closely linked to the ethical core of PCC, which emphasises recognising personhood and meeting persons as capable human beings rather than as passive recipients of care [22]. Prior research demonstrates that when patients receive tailored self‐management education, they actively adapt guidance to fit their everyday lives and circumstances, highlighting self‐management as a negotiated, context‐dependent process rather than passive compliance [41]. For HCPs, recognising invisible agency involves attending to patients' and family members' descriptions of everyday actions through open, person‐centred dialogue, allowing these efforts to be acknowledged and integrated into care planning [42, 43]. Furthermore, participants emphasised the importance of maintaining independence and living as close to their usual everyday lives as possible despite the wound. Recognising and supporting these everyday efforts is central to PCC's commitment to a meaningful life, which extends beyond functional outcomes and includes supporting patients' own goals and priorities [44, 45].

The participants appeared to move between different ways of engaging in care depending on the situation. This contrasts with previous research that has described patient involvement in more fixed terms, positioning patients as either active or passive [46]. In our study, what appeared to be more passive involvement was, in fact, situational and expressed in different ways. In some situations, patients described experiences that can be understood as being a bystander by choice, characterised by trust in HCPs and a sense of relief at handing over responsibility. Others described experiences that can be understood as bystander experiences by exclusion, marked by not being heard, receiving insufficient, inconsistent, or difficult‐to‐understand information, or lacking opportunities to influence their situation. These experiences risk being reinforced by clinical encounters that prioritise biomedical aspects over patients' concerns and priorities in care planning [47]. Another barrier to participation in care was insufficiently managed pain that limited patients' ability to process information, their willingness to undergo procedures and their agency in daily life. Given that pain is common among persons with hard‐to‐heal wounds [48], our findings highlight the importance of proactive pain assessment, addressing both daily pain and the need for anticipatory analgesia planning prior to wound care procedures, and clear communication when pain constrains patients' capacity for participation and informed consent. Educational approaches that develop advanced wound care competencies may strengthen HCPs' ability to assess and manage patients' pain and to communicate effectively about pain [49].

More broadly, the findings suggest that when patients' and family members' experiences, priorities, and efforts remain insufficiently recognised, this may reflect not only practical challenges in care planning but also broader conceptual challenges regarding how self‐management is understood and supported. This may partly stem from the lack of consensus on the definition of self‐management, which affects both research and clinical practice [23, 50]. This conceptual ambiguity contributes to variable practices, complicates outcome measurement, and hinders clinical efforts to identify which aspects of self‐management require support [23, 30]. A similar challenge can be identified within the field of PCC [51]. Person‐centred care is described and operationalised in multiple ways, with frameworks emphasising different aspects of care and operating at different levels of the healthcare system [21, 51, 52, 53, 54]. Although these frameworks offer valuable perspectives, the absence of a shared understanding of what constitutes PCC may create a gap between intention and implementation. In clinical practice, HCPs may perceive care as person‐centred, despite limited attention to patients' and family members' priorities, efforts and capacities [55]. At an organisational level, tools for assessing person‐centredness can therefore play an important role in making such gaps visible and supporting reflection and development towards more consistently enacted PCC [56].

Participants also described gaps in the care process, including delayed assessment and limited access to wound‐care expertise, which they perceived as contributing to prolonged healing. During these prolonged trial‐and‐error processes—characterised by uncertainty, delayed escalation, and inconsistent access to expertise—our analysis suggests that participants had a constrained sense of agency, with limited opportunities to influence the direction of care. Patients often became aware of what this had meant for their care only retrospectively, particularly when the care process changed, such as when more knowledgeable HCPs were involved. Reflections accompanied this retrospective understanding of the appropriateness of earlier care and their own role in the care process. This pattern can be further understood through Hackert et al. concepts of primary and secondary control [46]. When opportunities for primary control were constrained, participants appeared to rely more on secondary forms of control, such as adapting to the situation, enduring uncertainty and drawing on support from HCPs and family members to cope. This aligns with previous research emphasising the importance of supportive relationships in wound care [57]. An actionable implication of these findings is the need to strengthen opportunities for patients and family members to have primary control within the wound care pathway by introducing clearer, shared decision points. Developed and communicated together with patients and, when relevant, family members, such decision points could include planned reassessment at 2–4 weeks, explicit escalation triggers when healing does not progress as expected, and an agreed‐upon alternative plan if initial treatment proves ineffective [12]. Making these decision points explicit may reduce uncertainty, counteract stalled care trajectories and support patients and family members in maintaining an active role in the care process. In the Swedish context, care pathways and the national person‐centred wound care programme provide an important structural framework to support timely, systematic and aetiology‐based assessment and follow‐up, strengthening opportunities for more holistic and person‐centred wound care [3]. Complementing this, the Swedish National Registry for Ulcer Treatment (RUT), a national quality registry, supports HCPs in structured wound assessment, follow‐up and quality assurance in everyday practice, which has been associated with shorter healing times and reduced antibiotic use [14, 58, 59]. Together, national care pathways and registry‐based systems, such as RUT, may help mitigate unwarranted variation in PHC by supporting more standardised, evidence‐based wound care [60]. However, despite this evidence, the implementation of RUT varies considerably across Sweden. In the county where the present study was conducted, only a limited proportion of municipalities and PHC centres are connected to RUT [61], which constrains the conditions for systematic, timely and person‐centred wound care and points to an important area for development within PHC.

Patterns of shifting involvement and constrained agency were also evident among family members. Their involvement was characterised by responsibility without mandate: they performed meaningful tasks while simultaneously feeling uncertain and limited in their ability to act. This highlights gaps in the operationalisation of PCC, where family members' roles as partners in care are not always clearly recognised or supported [62, 63]. The findings highlight the need for more structured approaches to inviting and supporting family involvement. This may include seeking permission to involve family members, offering brief, targeted skills training when relevant and clarifying indicators of concern and appropriate points of contact if concerns arise. Approaches to involvement should remain sensitive to patients' and family members' varying wishes, capacities and circumstances, as not all wish or are able to take an active role in care [24]. To support HCPs in assessing whether and to what extent patients and family members wish to engage in wound care, Moore et al. model provides a structured approach to exploring preferences, capabilities and readiness for involvement [64]. Such approaches may also reduce the risk that heavily burdened family members remain invisible and unsupported within the care system [57].

Patients and family members accumulate valuable experiential knowledge of the wound care process, sharing experiences and offering ideas to improve wound care services. However, this experiential knowledge may remain insufficiently recognised within service development. Actively involving patients and family members—both in research and in local quality improvement initiatives within PHC—can help identify areas for improvement that might otherwise remain overlooked [24, 65, 66]. Although public and patient involvement in wound care research remains relatively limited, a previous study indicates that patients and family members wish to be involved, contribute their perspectives and take an active role in shaping wound care practices [65]. Advancing PCC in wound care, therefore, requires a system‐wide approach in which care is organised around partnership and supported by organisational structures and cultures that sustain PCC [67].

## 5 Strengths and Limitations

Credibility can be strengthened through crystallisation, understood as examining the same issue from multiple perspectives [68]. Accordingly, a strength of this study is its ability to capture the complexity of wound care as experienced by patients and family members across different care contexts, thereby enabling the exploration of diverse understandings of the care process. At the same time, the study has limitations. Although the findings provide valuable insights into family members' experiences, they are limited by the small number of participating family members. Recruitment through HCPs may have influenced which participants were reached. In addition, fewer patients were recruited from the municipal PHC, and no family members from this setting participated. However, perspectives on family members' roles and contributions were reflected in patients' accounts across care settings, providing indirect insight into family involvement.

Although these limitations should be considered, the findings are likely transferable to comparable regional and municipal PHC wound care settings. Organisational conditions, such as funding models, access to wound specialists, registry use and the extent to which PCC is embedded in practice may influence the identified dynamics.

## 6 Implications for Clinical Practice

The findings underscore the importance of making patients' and family members' existing knowledge and everyday efforts visible and of explicitly recognising them as meaningful contributions to care. Person‐centred care requires HCPs to routinely engage in conversations that explore what patients and family members already do to manage wound care in everyday life, the challenges they experience and how these efforts can be supported and integrated into care planning. By valuing and building on these contributions, clinicians may help clarify patients' and family members' roles in the care process, strengthen their sense of involvement and support safer, more sustainable wound care. This may also foster more trusting relationships in which patients and family members feel more confident raising concerns when something appears to be wrong.

The findings highlight the importance of organisational structures that support continuity, coordination and access to wound care expertise. Ensuring this is important for building trusting relationships, maintaining an overview of the wound care process, recognising changes over time and enabling timely escalation when healing does not progress as expected.

## 7 Implications for Further Research

Studies with larger, more diverse samples of family members are needed to advance knowledge of their circumstances and roles in wound care. Such research is important to deepen understanding of how they can be meaningfully integrated into care collaboration and how appropriate support can be developed, particularly given that their contributions may be overlooked when they are not physically present during healthcare encounters. Building on the current findings, future research should also examine variations in desired levels and forms of involvement among patients and family members in wound care and explore how PCC models can be developed and refined in collaboration with them in ways that are feasible within PHC and responsive to their needs.

## 8 Conclusion

This study advances understanding of the complex, dynamic process that patients and family members navigate in managing hard‐to‐heal wounds. One major finding is the invisible agency, wherein a person contributes meaningfully to care without always recognising their own role or potential. The findings emphasise the necessity of person‐centred wound care approaches that acknowledge and build on patients' and families' lived experiences, capacities and priorities. Achieving this requires collaborative, flexible care practices, supported by healthcare systems that provide timely access to wound‐care expertise and structured pathways. Although limited by sample size and setting, the study's insights inform future research and clinical efforts to enhance partnership‐driven wound care that is both effective and meaningful for those it serves.

## Funding

Post Graduate School for Integrated Care, Örebro University, with a grant from the Swedish Research Council (Vetenskapsrådet) (grant number 2022‐06319).

## Conflicts of Interest

The authors declare no conflicts of interest.

## Data Availability

The data that support the findings of this study are available on request from the corresponding author. The data are not publicly available due to privacy or ethical restrictions.
